# Histological and Gene Expression Analyses of the Arm and Finger Macroglands of Two *Hyloxalus* Frogs (Dendrobatidae)

**DOI:** 10.1111/mec.70162

**Published:** 2025-11-06

**Authors:** Diana Abondano Almeida, Marvin Anganoy‐Criollo, Taran Grant, Sofiia Klimovych, Lisa M. Schulte

**Affiliations:** ^1^ Department of Wildlife‐/Zoo‐Animal‐Biology and Systematics, Faculty of Biological Sciences Goethe University Frankfurt Frankfurt am Main Germany; ^2^ Departamento de Zoologia, Instituto de Biociências Universidade de São Paulo São Paulo Brazil

**Keywords:** dendrobatids, frogs, pheromones, RNA‐sequencing, sodefrin precursor‐like factors, specialised glands, dendrobátidos, ranas, feromonas, secuenciación del ARN, glándulas especializadas, Sodefrin Precursor‐like Factors

## Abstract

Chemical communication during courtship is well documented in salamanders and newts, but its role in frogs is less understood. In some Neotropical poison frogs, males exhibit specialised mucous glands (SMGs) in the hand integument that express high levels of sodefrin precursor‐like factors (SPFs), an amphibian pheromone. Some species also show integumentary swellings at the distal upper arm—known as the black arm gland (BAG)—of unclear function. We used histology and RNA sequencing to analyse the arm and finger integument of *Hyloxalus nexipus* and *H. azureiventris* to examine glandular composition, gene expression and potential pheromone production. We confirmed the co‐occurrence of two sexually dimorphic macroglands—swollen fingers and BAG—in *H. nexipus* , a rare trait in dendrobatids. Both structures differentially expressed SPF, suggesting complementary roles in courtship. We also provide the first histological characterisation of the BAG in *H. nexipus* and the homologous region in *H. azureiventris* , revealing that both are composed of specialised serous glands (SSGs). Notably, SPF expression in the BAG of *H. nexipus* indicates that SSGs, previously not linked to this function, can also produce proteinaceous pheromones. In *H. azureiventris* , no differential SPF expression was found in the arm, making its reproductive role uncertain. Both species expressed SPF in their fingers; thus, we hypothesise that *H. azureiventris* may have specialised glands despite lacking visible swelling, based on other dendrobatids where SPF is upregulated in male fingers with SMGs linked to pheromone production. Our findings reveal a novel pheromone‐producing gland and emphasise the complexity of chemical communication in dendrobatid reproduction.

## RESUMEN

La comunicación química durante el cortejo está bien documentada en salamandras y tritones, pero su papel en ranas es menos conocido. En algunas ranas venenosas neotropicales, los machos exhiben glándulas mucosas especializadas en la piel de la mano que expresan altos niveles de SPF, una conocida feromona sexual de los anfibios. Algunas especies también presentan hinchazones tegumentarias en la parte distal del brazo, conocidas como glándula negra del brazo, cuya función no ha sido determinada. En este trabajo, hemos utilizado la histología y la secuenciación de ARN para analizar el tegumento de los brazos y los dedos de *Hyloxalus nexipus* y *H. azureiventris* con el fin de examinar la composición glandular, los patrones de expresión génica y la posible producción de feromonas. Confirmamos la co‐ocurrencia de las dos macroglándulas sexualmente dimórficas (los dedos hinchados y la glándulas negras del brazo) en *H. nexipus*, un rasgo poco común en dendrobátidos. Ambas estructuras mostraron una expresión diferencial de SPF, lo que sugiere funciones complementarias en el cortejo. También proporcionamos la primera caracterización histológica de la glándula negra del brazo en *H. nexipus* y la región homóloga en *H. azureiventris*, y descubrimos que ambas están compuestas por glándulas serosas especializadas, que difieren morfológicamente entre especies. En particular, la expresión de SPF en la glándula negra del brazo de *H. nexipus* indica que las glándulas serosas especializadas, que hasta ahora no se habían relacionado con esta función, también pueden producir feromonas proteínicas. En *H. azureiventris* no se encontró expresión diferencial de SPF en el brazo, por lo que su función reproductora es incierta. Por el contrario, ambas especies expresaron SPF en los dedos, lo que sugiere que *H. azureiventris* también posee glándulas especializadas a pesar de carecer de hinchazón visible. En conjunto, nuestros hallazgos revelan nuevos tipos de glándulas productoras de feromonas y subrayan la complejidad de la comunicación química durante la reproducción en ranas dendrobátidas.

## 1 Introduction

Sexual chemical communication plays a pivotal role in the behaviour and interactions of many organisms. This mode of communication involves the release and detection of chemical signals, generally known as pheromones, which facilitate mate location, attraction, recognition and selection (Johansson and Jones 2007; Wyatt 2003, 2014). Among amphibians, salamanders and newts (Caudata) are well known for employing chemical communication as their primary mode of reproductive communication (Houck 2009; Woodley 2015). Key indicators of this communication mode include the occurrence of macroglands, the production and secretion of pheromones, and specific behaviours that facilitate their transmission and directly influence female behaviour (Houck 1998, 2009; Woodley 2015). Although often attributed only to males, recent evidence indicates that females can also possess courtship glands, potentially controlled by androgens (Gunelson et al. 2024). For instance, in *Desmognathus brimleyorum* , both sexes present such glands on the ventral tail surface, though they are larger in males than in females (Rollins and Staub 2017). In contrast, frogs (Anura) have traditionally been considered to rely primarily on acoustic and visual signals for communication during breeding (Amézquita and Hödl 2004; Arch and Narins 2009; Narins et al. 2003; Starnberger et al. 2014). However, recent studies have shown that frogs share similar traits with salamanders and newts, suggesting that chemical communication could also play an important role in this group during reproduction (Antoniazzi et al. 2022; Bossuyt et al. 2019; Willaert et al. 2013; Zheng et al. 2024).

Frogs possess specialised glands in the integument of secondary sexual characters such as nuptial pads (Duellman and Trueb 1994; Luna et al. 2018). These glands, commonly referred to as breeding glands or sexually dimorphic skin glands (SDSGs), develop and hypertrophy primarily during the breeding season (Brizzi et al. 2002, 2003; Thomas et al. 1993). They can form clusters known as macroglands, which are located in specific regions of the body, giving rise to structures such as abdominal glands, femoral glands, nuptial pads and axillary glands (Brizzi et al. 2003; Thomas et al. 1993). SDSGs have been recognised for their potential role in pheromone production and extensively studied across various amphibian species (Brunetti et al. 2015, 2016; Luna et al. 2018; Sever 2017; Thomas et al. 1993).

SDSG secretions can be volatiles or proteins that resemble known pheromones in other organisms (e.g., Schulte et al. 2023; Schulz et al. 2021) and are transmitted to females during courtship and breeding, making them a critical component of the male's reproductive strategy (Schulte et al. 2021). Examples of such compounds include volatile macrolides secreted from the femoral and gular glands of male mantellid and hyperoliid frogs, respectively (Kuhn and Schulz 2022; Melnik et al. 2019; Menke et al. 2016; Peram et al. 2017; Poth et al. 2013), and amplexins, proteins that are homologous to plethodontid modulating factor (PMF) and highly expressed in the nuptial pads of *Rana temporaria* and *Nidirana pleuraden* (Willaert et al., 2013; Zheng et al., 2024). Similarly, proteins of the two domain three‐finger proteins (2D‐TFP; e.g., sodefrin precursor‐like factor, phospholipase A2 inhibitor) are highly expressed in the male macroglands of several frog families including Hylidae, Pipidae, Nyctibatrachidae, Ranidae and Dendrobatidae (Abondano Almeida et al. 2024; Bossuyt et al. 2019; Schulte et al. 2021, 2022). SPF is a well‐known pheromone system in salamanders and newts (Houck et al. 2007; Janssenswillen, Willaert, et al. 2015; Maex et al. 2016; Van Bocxlaer et al. 2015) in which it has been shown to enhance female mating receptivity (Janssenswillen and Bossuyt 2016; Maex et al. 2018; Van Bocxlaer et al. 2015). This multigene pheromone protein family diversified through multiple gene duplications during the Late Palaeozoic, giving rise to two ancient clades: alpha and beta SPFs (Janssenswillen, Vandebergh, et al. 2015; Van Bocxlaer et al. 2015). The specific SDSGs associated with the production of these proteins are the specialised mucous glands (SMGs), which are larger than ordinary mucous glands (OMGs) and contain tall columnar cells in their secretory portion (Luna et al. 2018). While SPF expression can occur in non‐glandular tissues, male macroglands typically exhibit a significantly higher diversity of SPF precursors compared to non‐reproductive tissues (Janssenswillen, Willaert, et al. 2015).

Another type of SDSG is the specialised serous glands (SSGs), characterised by a syncytial internal secretory layer and a lumen filled with granules (Luna et al. 2018). SSGs are less frequent in male macroglands, with some examples found in the femoral glands of male mantellid frogs (Luna et al. 2018; Vences et al. 2007) and the ventrolateral glands of several hylid species (Brunetti et al. 2015; Luna et al. 2019) that come into direct contact with the female's dorsum during axillary amplexus (Campbell and Smith 1992; Thomas et al. 1993). The femoral glands of mantellids secrete volatile macrolides that play a role in male–male interactions (Poth et al. 2012) and potentially in reproductive contexts through direct contact with the female's shoulder during modified amplexus (Blommers‐Schlösser 1975, 1979). Similarly, Schulte et al. (2024) reported high expression of nicotinamide N‐methyltransferase (NNMT)—an enzyme in the class I‐like SAM‐binding methyltransferase superfamily—in the male ventrolateral glands containing SSGs of the hylid *Ptychohyla macrotympanum* . In the same study, they found that NNMT‐like genes exhibit striking diversity in anurans, particularly in frogs and salamanders, suggesting a reproductive role (Schulte et al. 2024). In contrast, Schulte et al. (2024) found that the expression of SPF transcripts in ventrolateral glands was relatively low compared to other genes including NNMT.

Interestingly, different types of male macroglands can co‐occur within the same individual, each potentially playing complementary roles in frog biology and behaviour (Brunetti et al. 2015; Luna et al. 2019). For example, some species of the hylid genus *Plectrohyla* possess both nuptial pads and swollen upper lips (for review, see Luna et al. 2019). Additionally, different specialised glands can be present in distinct macroglands, as seen in *Ptychohyla*, in which SMGs are found in nuptial pads and SSGs in the ventrolateral glands (Luna et al. 2019). To date, only one study has focused on the gene expression of multiple breeding glands within the same individual. Schulte et al. (2024) examined the macroglands of *P. macrotympanum* males, noting largely different gene expression patterns in the SMGs in the nuptial pads and SSGs in the ventrolateral glands. However, the potential functions of these two types of macroglands in this species still remain unknown.

Another intriguing and more promising model system to study the evolution and possible function of co‐occurring macroglands is the Neotropical poison frogs of the superfamily Dendrobatoidea (Dendrobatidae and Aromobatidae; sensu Grant et al. 2006). Adult males of many poison frog species exhibit ‘swollen fingers’, caused by hypertrophied SMGs in finger IV (and, less frequently, the wrist and other fingers as well), a characteristic known exclusively in this group, only in males (Cavalcanti et al. 2022; Dunn 1924; Grant et al. 2006). A recent study reported high expression of SPF transcripts in the swollen fingers of two dendrobatid species, *Leucostethus brachistriatus* and *Epipedobates anthonyi* , suggesting a potential role of these glands in sexual chemical communication (Abondano Almeida et al. 2024). However, some species not only develop SMGs in their fingers, but additionally another type of sexually dimorphic swelling on the arm, referred to as the ‘black arm band’ or ‘black arm gland’ (BAG; Grant and Castro‐Herrera 1998; Grant et al. 2006, 2017). The BAG comprises a region of swollen, usually heavily melanised (grey or black) skin along the ventral and medial surfaces of the distal end of the upper arm, often extending along the medial surfaces of the proximal lower arm. It is known in only a few species of the dendrobatid genus *Hyloxalus* (Grant and Castro‐Herrera 1998, Grant and Ardila‐Robayo 2002, Grant et al. 2006, 2017, Acosta‐Galvis et al. 2020). Adult females and juveniles of those species lack the BAG, and the coloration and the swelling in the adult males vary from indistinct to conspicuously hypertrophied (Grant and Castro‐Herrera 1998, Grant et al. 2006, 2017). Given its restriction to adult males and variation in size, Grant and Castro‐Herrera (1998) proposed that it could be analogous to the swelling of finger IV, with ‘both characters under hormonal control and used in amplexus’ (Grant and Castro‐Herrera 1998). The recent finding of the high expression of SPF in the swollen fingers of dendrobatid frogs suggests that these species might also express putative pheromones in the BAG.

**FIGURE 1:**
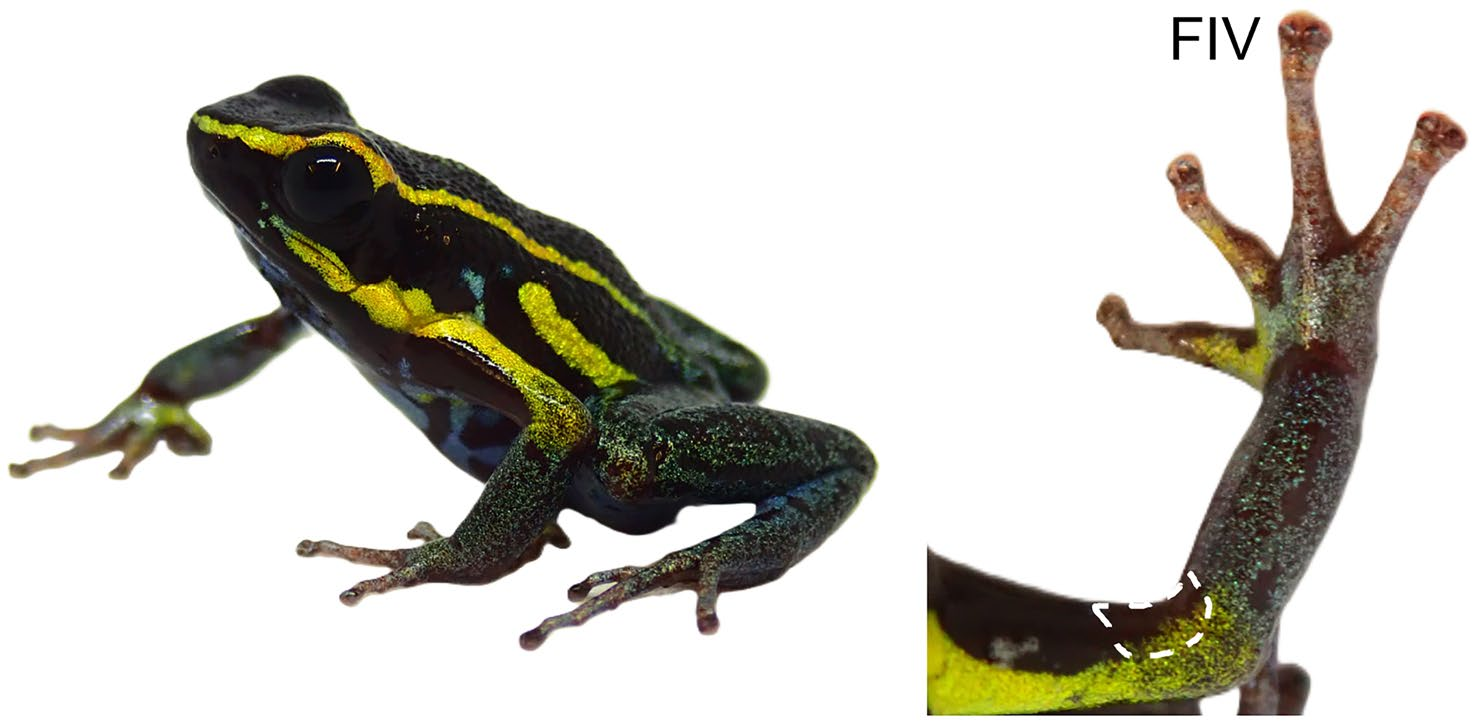
Male of the dendrobatid frog *Hyloxalus azureiventris* (left). Dorsal view of the arm highlighting the fourth finger (IV) and the region homologous to the BAG (outlined with a dashed white line; right). Photo: Christopher Heine.

**FIGURE 2:**
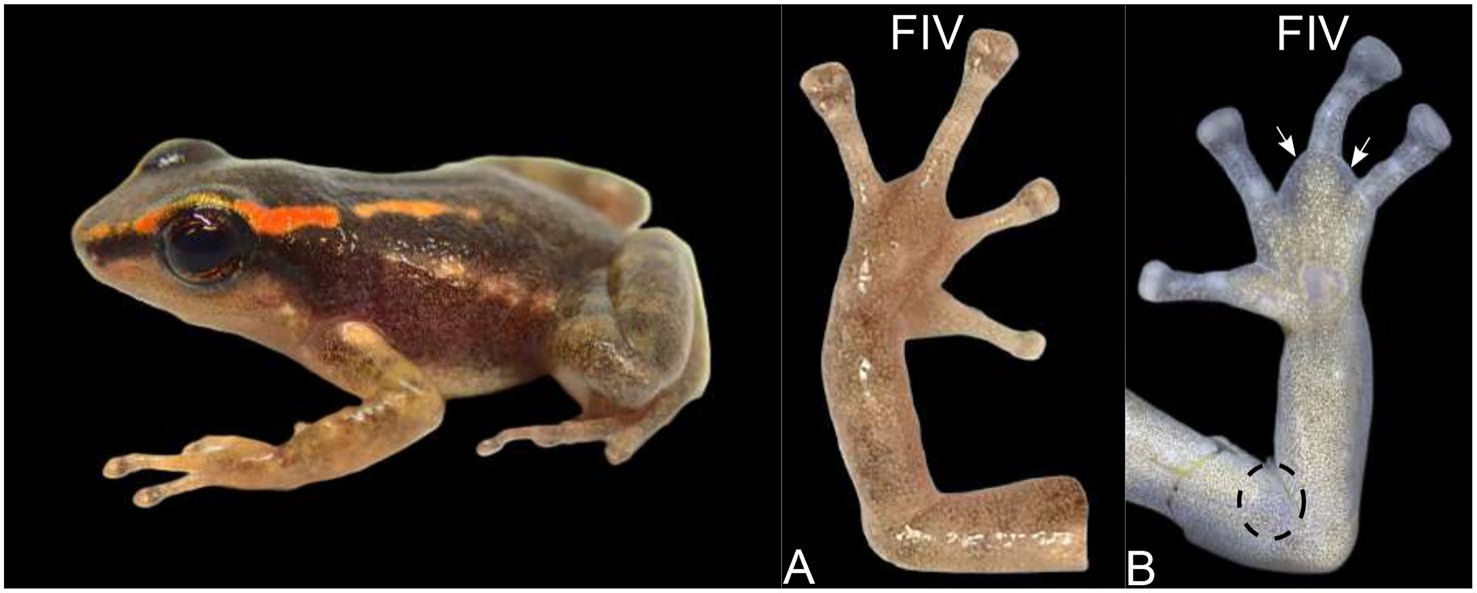
Male of the dendrobatid frog *Hyloxalus nexipus* (left). (A) Dorsal view of the arm of a captive‐reared male, showing no swelling in finger IV and no grey or black pigmentation in the BAG region. (B) Ventral view of the arm of a wild‐caught male, showing pronounced swelling in the base of finger IV (indicated by arrows) and visible BAG pigmentation, with the region circled by a dashed black line. Photos: Whole‐body and panel (A) by Christopher Heine; panel (B) by Marvin Anganoy‐Criollo.

Our study aims to investigate and compare the glandular composition and role in pheromone production of the integument on the arms and fingers of males from two non‐closely related *Hyloxalus* species: *Hyloxalus azureiventris* (Kneller and Henle, 1985) and *Hyloxalus nexipus* (Frost 1986). Males and females of *H. azureiventris* (Figure 1) have been reported to, at least externally visible, lack both the BAG and swelling of the fingers and wrist (Grant et al. 2006, 2017), and lack cephalic amplexus (Lötters et al. 2007), a behaviour that has been suggested to be important for pheromone transmission in species with elevated SPF expression in their swollen fingers (Abondano Almeida et al. 2024). In contrast, males from *H. nexipus* (Figure 2) display both the BAG and basal swelling on finger IV, the latter containing SMGs (Cavalcanti et al. 2022); females of this species lack SMGs and BAG (Grant et al. 2017; Cavalcanti et al. 2022). The co‐occurrence of BAG and swollen fingers is rare (Cavalcanti et al. 2022), providing a valuable opportunity to study different gland types and gene expression patterns in the co‐occurrence of macroglands and their role in pheromone production. This research combines histology, whole‐transcriptome sequencing (RNA‐seq), and differential gene expression analysis to explore and compare male glandular tissue composition, gene expression patterns and potential pheromone production. We aim to advance our knowledge of the molecular basis of glandular function in the male integument and its role in amphibian reproduction.

## 2 Materials and Methods

### 2.1 Biological Sample Collection

We sampled males from *H. azureiventris* and *H. nexipus* , both from a lab‐reared population kept at Trier University in Trier, Germany since 2020. The population of both species originates from near Tarapoto, Peru. We sampled six *H. azureiventris* individuals (four individuals for RNAseq and two for histological examinations) and four *H. nexipus* (all for RNAseq). The sampled individuals had previously reproduced and were housed in mixed‐sex enclosures, although their reproductive activity on the day of sampling is unknown. We did not observe any swelling in the fingers of any of the individuals of *H. azureiventris* , nor on the ventral and anterior skin surface at the end of the upper arm, although this region was hypermelanised (Figure 1). Similarly, we did not observe any swelling in the fingers and arm, nor any grey or black coloration on the BAG area of the lab‐reared specimens of *H. nexipus* (Figure 2A).

We also studied the histology of a wild‐caught adult male *H. nexipus* obtained from the Herpetological Division of the University of Kansas Museum of Natural History (Table S1). Cavalcanti et al. (2022) corroborated the presence of finger swelling and SMGs in this specimen, and we observed pale grey swelling consistent with the BAG (Figure 2B). The histological examination of *H. nexipus* was done at the Universidade de São Paulo, São Paulo, Brazil, and that of *H. azureiventris* at Goethe University, Frankfurt am Main, Germany.

The frogs were euthanised by an initial inhalation of 4% isoflurane, followed by an injection of tricaine methanesulphonate (100 mg/kg dose). Immediately afterward, we removed the target tissue (i.e., whole finger IV and distal end of the upper arm or BAG area) and control tissue (i.e., whole toe IV and the analogous region of the BAG, the distal upper leg including the ventral and medial surfaces, hereafter referred to as leg skin) for RNA sequencing analysis. The samples from the same individual and same tissue were pooled and preserved in *RNAlater* (Life Technologies) in a refrigerator at ca. 4°C overnight and then in a freezer at ca. −20°C until RNA extraction.

### 2.2 Histology

We conducted a histological analysis to confirm the presence or absence of specialised glands in the integument of the distal end of the upper arm in both species. We excluded the histology of finger IV of *H. nexipus* because it has been studied previously (Cavalcanti et al. 2022). Methodological errors in the histological procedure prevented us from obtaining results for the fingers of *H. azureiventris* .

The tissue around the arm was fully removed (including the BAG), cutting laterally, and pulled off from the arm, dividing this ‘sleeve’ into dorsal and ventral areas, approximately 15 mm^2^ each of the species. We cut the target tissue into a large section, ensuring the BAG area remained intact for analysis. We took as a control the tissue from the skin near the elbow area on the dorsal side (proximal and distal to the elbow), where no glands are expected. Samples of both species were dehydrated in a graded series of isopropanol (70%–100%); after, the skin of *H. azureiventris* was embedded in HEMA (2‐hydroxyethyl methacrylate), Technovit 7100, while the sample of *H. nexipus* was embedded in methacrylate glycol resin (Historesin Leica). The tissue of each species was cut at 4–5 μm with a rotary microtome (diamond blade) mounted onto microscope slides, and stained with Coomassie Brilliant Blue R‐250 (CBB) for protein detection (Kiernan 2003; for *H. azureiventris* ) and with and toluidine blue with basic fuchsin staining to visualise the general structure (for *H. nexipus* ). For both species Periodic acid–Schiff (PAS) was used for the detection of mucins, glycogens and neutral glycoproteins (Lillie 1947; McManus 1946; Mowry 1963). We carried out the histological analyses with a Leica DM LB2 and a DM 750 microscope. We captured images of the sections with a Bresser MikroCam II 5MP HIS and Leica ICC50 W camera and the software MicroCamLab and Leica DM 750, with a built‐in camera Leica ICC50 W. Characterisation of the integument, exocrine glands and specialised glands followed the criteria of Cavalcanti et al. (2022) and Luna et al. (2019).

### 2.3 RNA Extraction, Library Preparation and Sequencing

We followed the method of Abondano Almeida et al. (2024) for RNAseq analysis. For each male, we sampled the integument of the distal end of the upper arm, equivalent to the BAG area, and finger IV as target tissues, with the leg skin and toes serving as controls. From the same individual we sampled the right and left integument of the distal end of the upper arm, leg skin, fingers and toes. The samples were pooled according to tissue type (e.g., integument of the distal end of the upper arm from left and right arm together). Overall, a total of 32 samples were sequenced for this study in a paired‐sample design (i.e., paired finger–toe, and integument of the distal end of the upper arm–leg skin tissue samples for each of the four males per species). The RNA extraction was done using the RNeasy Plus Universal Mini Kit (Qiagen) at Goethe University, Frankfurt am Main, Germany.

RNA extractions were sent to Genewiz in Leipzig, Germany for processing, where they assessed RNA quantity and quality, prepared sequencing libraries and performed sequencing. The RNA concentration was measured using a Qubit 4.0 Fluorometer (Life Technologies, Carlsbad, CA, USA), and RNA integrity was evaluated with an RNA Kit on an Agilent 5600 Fragment Analyser (Agilent Technologies, Palo Alto, CA, USA). The RNA Quality Number (RQN) ranged from 9.9 to 10 in all samples. RNA sequencing libraries were prepared using the NEBNext Ultra II RNA Library Prep Kit for Illumina according to the manufacturer's protocol (New England Biolabs, Ipswich, MA, USA). This process involved initial enrichment of mRNAs with Oligod(T) beads, followed by fragmentation of the enriched mRNAs for 15 min at 94°C. First‐strand and second‐strand cDNA synthesis followed, with end‐repair and 3′‐adenylation of cDNA fragments. Universal adapters were then ligated to the cDNA fragments, followed by index addition and library enrichment by PCR with limited cycles. The sequencing libraries were validated using the DNA Kit high sensitivity DNA 1–6000 bp from Agilent on the Agilent 5600 Fragment Analyser (Agilent Technologies, Palo Alto, CA, USA), and quantified with the Qubit 4.0 Fluorometer (Invitrogen, Carlsbad, CA).

The sequencing libraries were multiplexed and clustered onto a flow‐cell on the Illumina NovaSeq 6000 according to the manufacturer's guidelines. Sequencing was performed using a 2 × 150 bp paired‐end (PE) configuration v1.5. Image analysis and base calling were carried out by NovaSeq Control Software (NCS) v1.7. on the NovaSeq Instrument. Raw sequence data (bcl files) from the Illumina NovaSeq were converted to fastq files and de‐multiplexed using Illumina bcl2fastq v.2.20 software, allowing one mismatch for index sequence identification. Raw sequencing reads are available from the European Nucleotide Archive under project accession number PRJEB102072.

Adaptor sequences and low‐quality bases were trimmed with Trim Galore v.0.5.0 (Krueger 2016), discarding reads that were trimmed to < 24 bp in length and keeping unpaired reads. Additional trimming was performed with Trimmomatic v.0.38 (Bolger et al. 2014), retaining sequences of at least 25 bp, with an average quality (phred33) > 20, then trimming along a sliding window of 4 bp and removing bases below quality 15, and finally removing leading and trailing bases below quality 3. Reads were corrected for random Illumina sequencing errors using Rcorrector v.1.0.3.1 (Song and Florea 2015).

### 2.4 Assembly

Our assembly approach included (1) assembly on an individual basis using both tissue samples (target and control) simultaneously. Paired samples consisted of fingers and toes, and integument of the distal end of the upper arm and leg tissues. For each pair, both tissues were analysed together, with one designated as the target (e.g., fingers or integument of the distal end of the upper arm) and the other designated as the control (e.g., toes or leg skin); (2) performing each assembly at multiple kmer sizes; and (3) merging the multiple assemblies into a single non‐redundant assembly for each species. This multi‐kmer assembly strategy, followed by merging with the tr2aacds pipeline (Gilbert 2013), is known to produce assemblies with greater completeness and reduced sequence duplication, with respect to BUSCO benchmarks (Mamrot et al. 2017). For each individual, we used kmer sizes of 25, 53 and 75 in SPAdes v.3.12 (Bankevich et al. 2012) incorporating both sets of reads (arm and leg skin; fingers and toes). With four individuals sequenced per species, this resulted in a total of 12 assemblies for each tissue type per species. For *H. nexipus* , only 11 assemblies were produced for finger and toe samples due to computational limitations preventing the use of kmer 25. The individual‐based assemblies were then merged into a single species‐level assembly using the EvidentialGene (Evigene) tr2aacds pipeline v.2017.12.21 (Gilbert 2013). The completeness of these merged assemblies was evaluated with BUSCO v.3.0.2 (Simão et al. 2015) using the vertebrata_odb9 database and contig metrics were obtained using TransRate v1.0.125 (Smith‐Unna et al. 2016). All read and assembly metrics are detailed in Tables S2–S9. It is important to highlight that ours is a short‐read based assembly that has not been verified by longer read methods (e.g., PCR/Sanger, PacBio, Nanopore).

### 2.5 Annotation, Read Alignment and Gene Expression Analysis

For each species and paired sample, we annotated the final merged (evigene) assembly with Diamond v.0.9.22.123 (Buchfink et al. 2015), using Blastx mode to search assembly transcripts against the Identical Protein Groups database from NCBI (2024) including only vertebrate sequences. For assembly and read alignment statistics results refer to Tables S2–S9. Sequencing reads were aligned to the reference transcriptome using HISAT2 version 2.1.0 (Kim et al. 2015) Reads aligning to annotated transcript regions were counted using FeatureCounts version 2.0.6 (Liao et al. 2014), which provided the data for differential expression analysis. Assemblies and annotations are available on Dryad (https://doi.org/10.5061/dryad.vq83bk45g).

The differential expression (DE) analysis was conducted with the R package ‘DESeq2’ (version 4.2). The Benjamini‐Hochberg correction was applied to *p*‐values to control for multiple hypothesis testing (Love et al. 2014). Given our paired‐sample design (integument of the distal end of the upper arm and leg skin or fingers and toes), the final model included tissue type as a fixed effect and individual as a random effect to account for both treatment and individual variation. We visualised DE transcripts and the magnitude and statistical significance of expression changes using a Volcano plot created with the R package ‘EnhancedVolcano’ (Blighe et al. 2018; version 1.16.0). Differentially expressed genes were identified with an FDR *p*‐value threshold (*p* < 0.05) and a magnitude of differential expression (log_2_ fold change) ≥ 0. Among the filtered DE transcripts, we identified those predicted to encode extracellular proteins (indicative of secretion from gland cells) using DeepLoc v.2.0 (Thumuluri et al. 2022) or having a signal peptide (suggestive of involvement in protein secretion) using SignalP v.6.0 (Teufel et al. 2022). We also manually checked annotations for potential pheromone functions and examined transcripts known to be significant or highly expressed in other frogs' glands or reproductive systems. The list of annotated genes and differential expression analysis from both species is presented in Table S10.

## 3 Results

### 3.1 Histology

#### 3.1.1 Black Arm Gland

**FIGURE 3:**
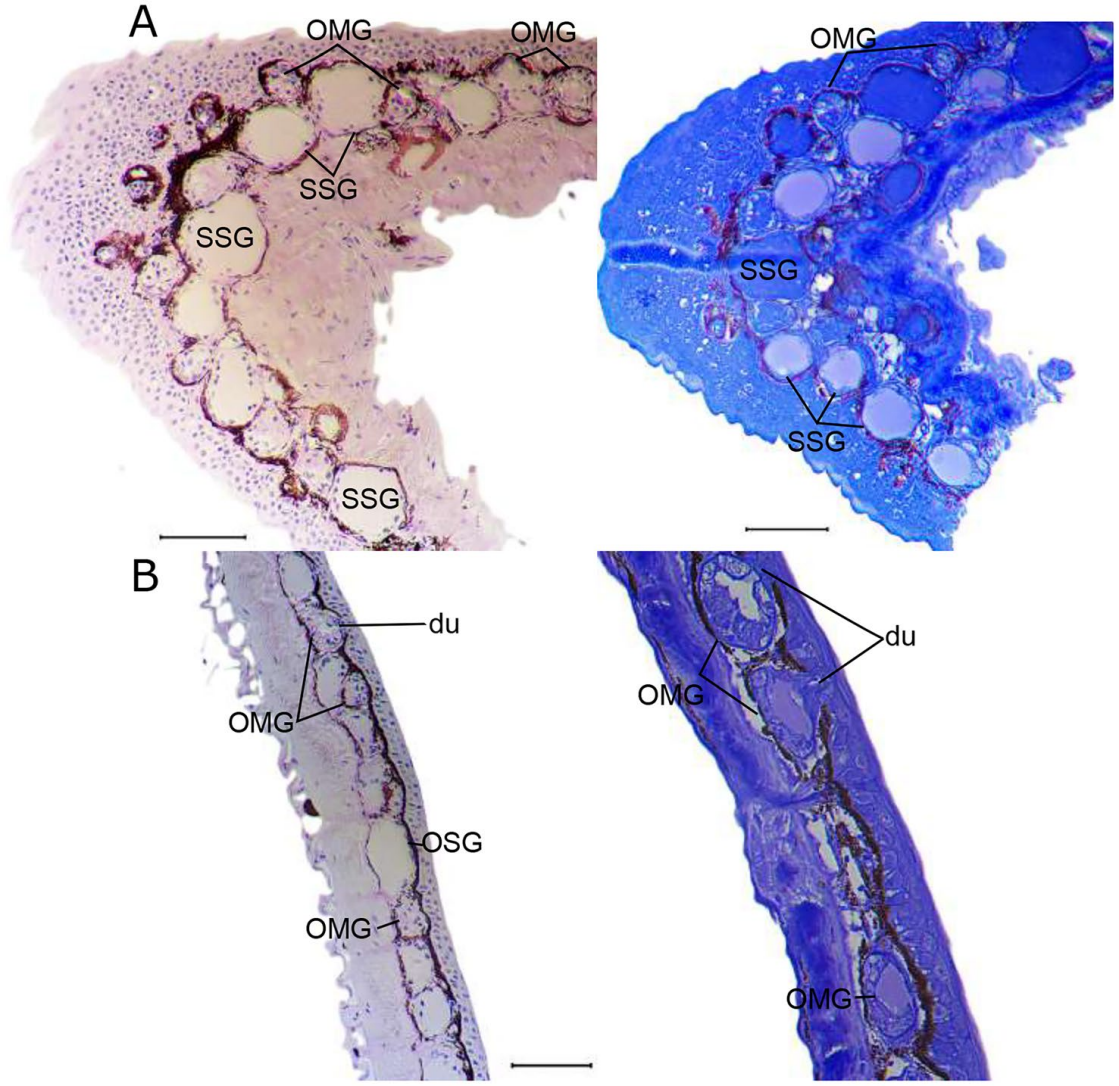
Histological sections of adult male integument from *Hyloxalus azureiventris* . (A) Distal end of the upper arm (homologous region of the BAG). (B) Dorsal side proximal and distal to the elbow. Left: Periodic acid‐Schiff staining, Right: Coomassie Blue R‐250 staining. du, duct; OMG, ordinary mucous gland; SSG, specialised serous gland. Scale bars 100 μm.

The integument of the anteroventral skin on the distal end of the upper arm of *H. azureiventris* (equivalent to the region of the BAG) is composed of 5–7 epithelial cell layers on the epidermis. Despite the lack of externally visible integumentary swelling, the stratum spongiosum is a wide space containing a few ovoid OMGs and several large, ovoid SSGs arranged in one or sometimes five layers; some SSGs are surrounded by a thin layer of melanophores. The SSGs are 2–4 times larger than the OMGs and OSGs (ordinary serous glands) of the dorsal elbow area (description below); the syncytium is homogeneous, and no granules are visible. SSGs are enveloped by a myoepithelial sheath with basal nuclei that are rounded. The stratum compactum is approximately the same thickness as the stratum spongiosum. The OMGs are slightly CBB‐ and PAS‐positive, whereas the SSGs are CBB‐positive, varying from pale to intense blue (confirming the presence of proteins) and PAS‐negative (Figure 3).

The male dorsal integument near the elbow (control tissue) of *H. azureiventris* has 2–3 epithelial cell layers in the epidermis. In the dermis, the stratum compactum is approximately the same thickness as the stratum spongiosum. The melanophores are located in the stratum spongiosum and form a thin, discontinuous layer between the epidermis and the stratum compactum, covering the glands. The secretory portion is ovoid or elongated in the OMGs and ovoid in the OSGs. These glands are separate and interleaved, forming a discontinuous or continuous monolayer in a ratio OMG 2:1 OSG. Both OMGs and OSGs are slightly CBB‐positive, whereas the OMGs are PAS‐positive while the OSGs are PAS‐negative (Figure 3).

**FIGURE 4:**
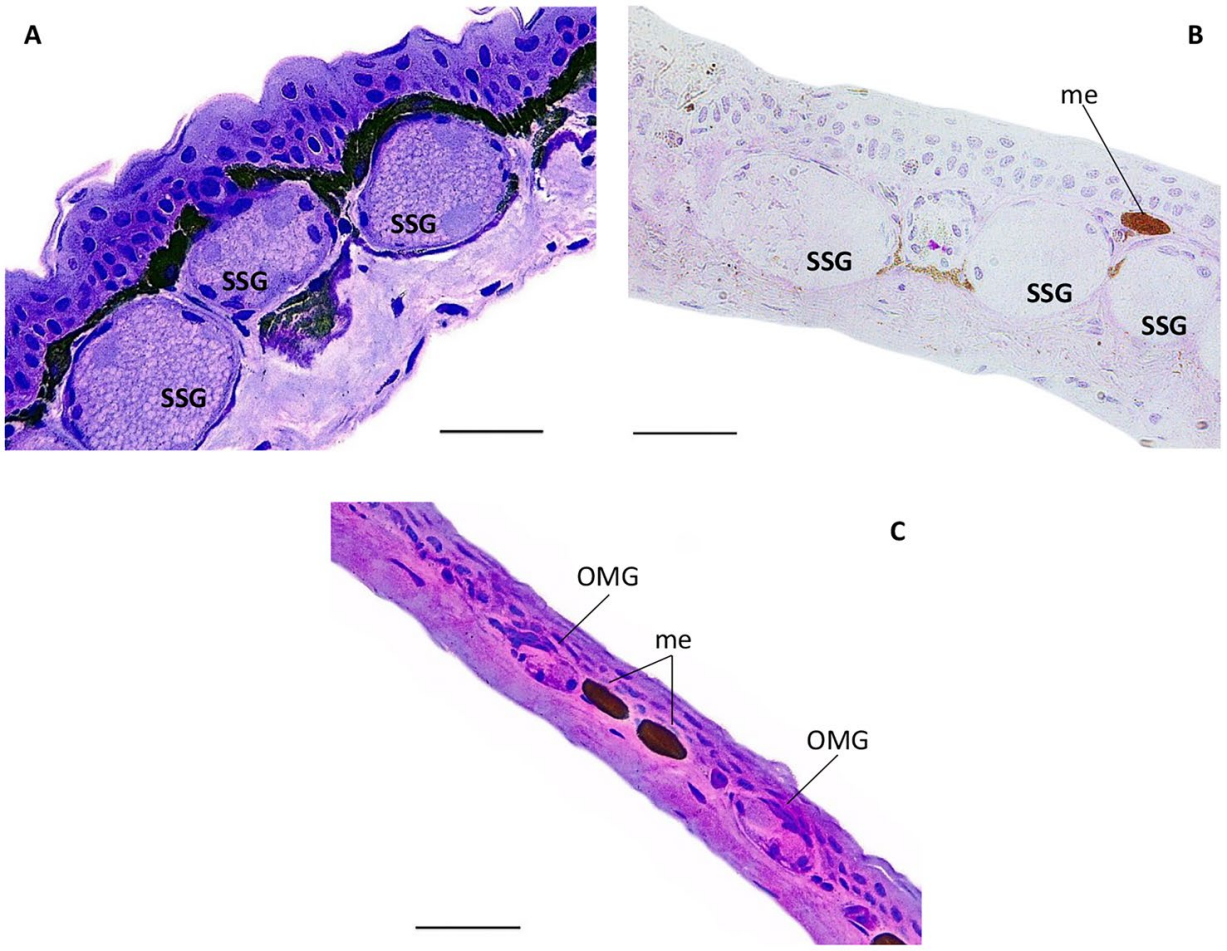
Histological sections of adult male from *Hyloxalus nexipus* . (A, B) Black arm gland, (C) dorsal integument of the elbow area of the adult male *H. nexipus*. (A, C) Toluidine blue with basic fuchsin staining, (B) periodic acid‐Schiff staining. Me, melanophores; OMG, ordinary mucous gland; OSG, ordinary serous gland; SSG, specialised serous glands. Scale bars 100 μm.

As noted above, male *H. nexipus* possesses pale grey swelling of the ventral and anterior integument along the distal portion of the upper arm (i.e., a BAG). The integument of this macrogland is composed of an epidermis with three epithelial cell layers, a thin layer of the melanophores between the epidermis and stratum spongiosum, and a stratum spongiosum containing a few OMGs and many rounded large serous glands arranged in a monolayer. Like *H. azureiventris* , the SSGs are two to three times larger than both the adjacent OMGs and the OSGs of the elbow area (description below), but the syncytium of SSGs is granular with homogeneous rounded regions. The SSGs are enveloped by a myoepithelial sheath with rounded basal nuclei. The stratum compactum is half as thick as the stratum spongiosum, and there are 3–5 more SSGs than OMGs. OMGs and SSGs stained PAS negative (Figure 4).

The dorsal integument of the elbow area of the adult male *H. nexipus* is similar to that of *H. azureiventris* , except that there are a few separate melanophores between the epidermis and s. compactum, without covering the glands, and OMGs and OSGs are well separated from each other. There are 2:1 OMGs to OSGs (Figure 4).

### 3.2 Differential Expression of Sodefrin Precursor‐Like Factor and Other Proteins of Interest

#### 3.2.1 Black Arm Gland

**FIGURE 5:**
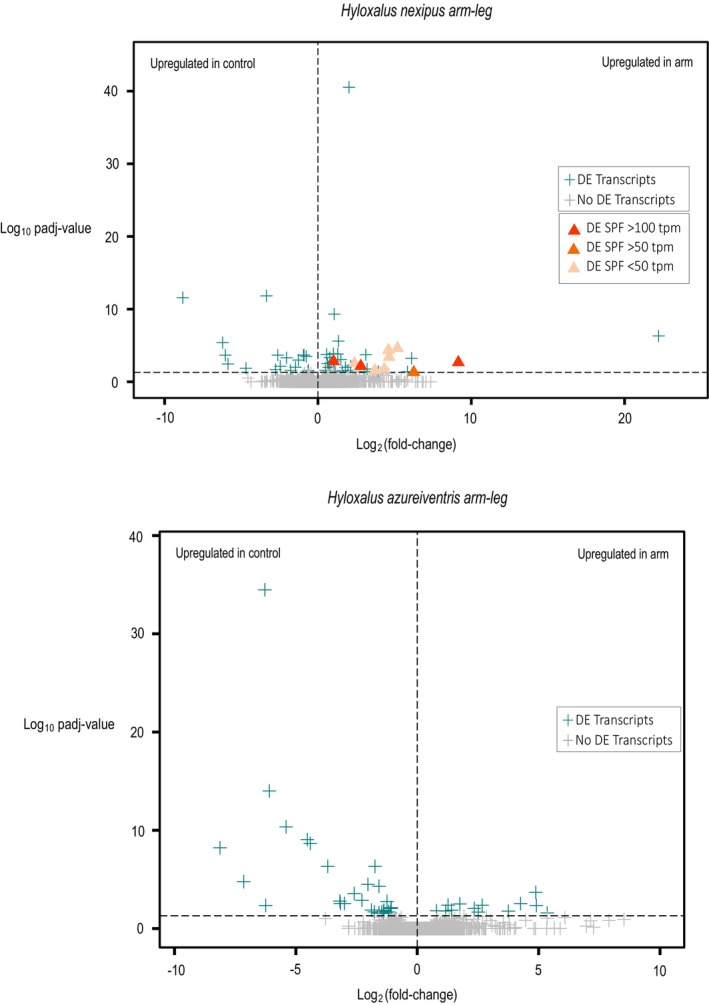
Volcano plots illustrating the differential expression analysis between arm (target) and leg (control) tissues in *Hyloxalus nexipus* (top) and *H. azureiventris* (bottom). Each transcript is represented by a symbol, with the *x* axis indicating the magnitude of change (log_2_ fold change) and the *y* axis showing the statistical significance (−log_10_ adjusted *p*‐value). Differentially expressed (DE) transcripts are shown in cyan, while non‐significant transcripts are depicted in grey. DE transcripts coding SPF genes are highlighted as orange triangles (▲), with varying intensities of orange reflecting their expression levels in transcripts per million (TPM). All other transcripts are shown as plus signs (+).

In *H. nexipus* , 65 of the 43,582 annotated transcripts were differentially expressed in the BAG region compared to the leg (Table 1). Among these 65 DE transcripts, ten encoded SPFs, all upregulated and with a log_2_ Fold Change > 1 including six of the top ten transcripts most strongly upregulated in the BAG (Figure 5). Additionally, another 93 transcripts of the two domain three‐finger family proteins (2D‐TFP; viz. sodefrin precursor‐like factor, phospholipase A2 inhibitor) were not differentially expressed. No putative amphibian pheromones other than SPF were differentially expressed in the BAG of *H. nexipus* . However, we also detected several highly expressed transcripts that have also been reported in the breeding glands of several male breeding glands of several frog families and could be related to amphibian reproduction, including lysozyme C, cysteine‐rich venom protein and ovostatin, kunitz‐type serine protease, all of which are secretory, extracellular proteins (Table 3). Other differentially expressed transcripts were uromodulin, and di‐N‐acetylchitobiase, both secretory and extracellular proteins (Table S10). Their role, if any, in amphibian reproduction is unknown.

**TABLE 1:** Summary of the differential gene expression analysis (DE) of the paired sample arm and leg tissue.

Arm and leg tissue	*Hyloxalus azureiventris*	*Hyloxalus nexipus*
Total transcripts in assembly	51,473	43,582
DE transcripts (*p*_adj_ value < 0.05)	44	65
Transcripts upregulated in arm	14	45
Transcripts downregulated in arm	30	20
Total SPFs in assembly	47	103
Upregulated SPF in arm (P_adj_ value < 0.05)	0	10
Downregulated SPF in arm	0	0

In *H. azureiventris* , 44 of the 51,473 annotated transcripts were differentially expressed in the arm integument compared to the leg (Table 1, Figure 5). None of these encoded SPFs or other putative amphibian pheromone‐related proteins. The only upregulated transcript that has also been reported as upregulated in the breeding glands of other frogs was a serine protease inhibitor (a non‐secretory extracellular protein; Table 3). Additionally, serotransferrin and cystatin—both extracellular proteins—were upregulated, with cystatin being a commonly found component of social transfer materials in vertebrates (Hakala et al. 2023; Table 3). Interestingly, several transcripts encoding NNMT and one encoding trefoil factor were downregulated; NNMT proteins have been found to be highly expressed in sexually dimorphic skin glands in the male ventrolateral glands of *P. macrotympanum* (Schulte et al., 2024) and have shown both up‐ and down‐regulation in the swollen fingers of two dendrobatid species (Abondano Almeida et al. 2024).

#### 3.2.2 Finger IV

**FIGURE 6:**
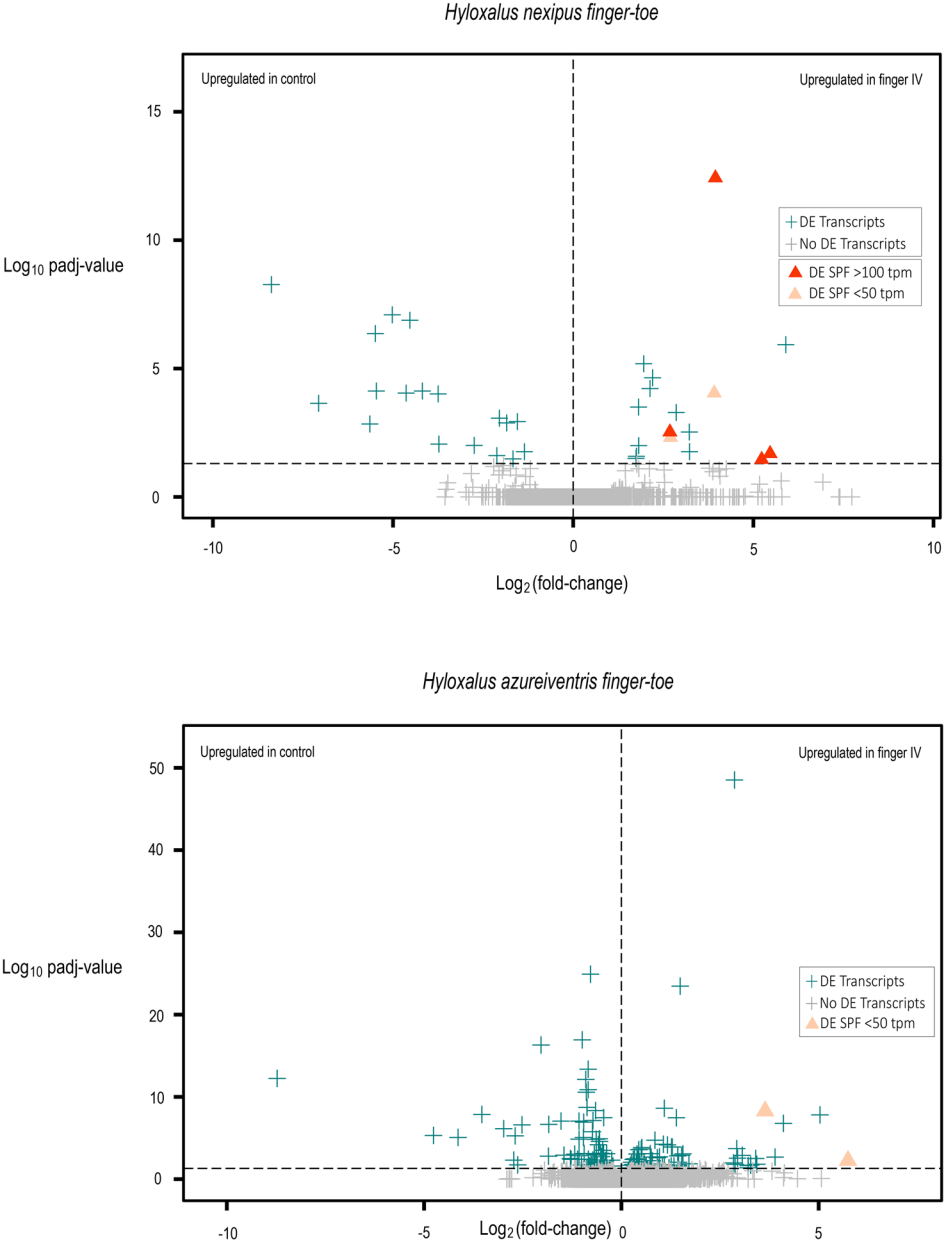
Volcano plots illustrating the differential expression analysis between fingers (target) and toes (control) tissues in *Hyloxalus nexipus* (top) and *H. azureiventris* (bottom). Each transcript is represented by a symbol, with the *x* axis indicating the magnitude of change (log_2_ fold change) and the *y* axis showing the statistical significance (−log_10_ adjusted *p*‐value). Differentially expressed (DE) transcripts are shown in cyan, while non‐significant transcripts are depicted in grey. DE transcripts coding SPF genes are highlighted as orange triangles (▲), with varying intensities of orange reflecting their expression levels in transcripts per million (TPM). All other transcripts are shown as plus signs (+).

In *H. nexipus* , 37 of the 63,325 annotated transcripts were upregulated in the finger IV compared to the toe IV (Table 2). Of these, six encoded for two domain three‐finger family proteins (2D‐TFP; viz. sodefrin precursor‐like factor, 5 transcripts; phospholipase A2 inhibitor, 1 transcript) that were included in the ten most upregulated transcripts in the swollen fingers (Figure 6), all with a log_2_FC > 2; 99 other transcripts annotated as 2D‐TFP (viz. sodefrin precursor‐like factor, phospholipase A2 inhibitor) were not differentially expressed. In *H. azureiventris* , 156 of the 48,740 annotated transcripts were differentially expressed in the finger IV compared to the toe IV (Table 2) and only two upregulated DE transcripts encoded SPF (Figure 6); 81 other transcripts annotated as 2D‐TFP (viz. sodefrin precursor‐like factor, phospholipase A2 inhibitor) were not differentially expressed. No other putative amphibian pheromone was differentially expressed in either species' fingers.

**TABLE 2:** Summary of the differential gene expression analysis (DE) of the paired sample fingers and toes.

Fingers and toes	*Hyloxalus azureiventris*	*Hyloxalus nexipus*
Total transcripts in assembly	48,740	63,325
DE transcripts (*p*_adj_ value < 0.05)	156	37
Transcripts upregulated in fingers	77	18
Transcripts downregulated in fingers	79	19
Total SPFs in assembly	83	105
Upregulated SPF in fingers (P_adj_ value < 0.05)	2	6
Downregulated SPF in fingers	0	0

Furthermore, we detected two upregulated transcripts in the swollen fingers of *H. nexipus* that are also highly expressed in the male macroglands of several frog families, namely ranaspumin (a secretory, extracellular protein) and nicotinamide N‐methyltransferase (NNMT, non‐secretory, intracellular protein; Abondano Almeida et al. 2024; Schulte et al. 2024). Additionally, we observed high expression of prostaglandin reductase and ADP‐ribosylation factor (Table S11).

In the fingers of *H. azureiventris* , we identified several differentially expressed transcripts that are also highly expressed in male breeding glands of other frog species. These included pancreatic lipase (a secretory and extracellular protein), ranaspumin (a secretory and extracellular protein), ovostatin (a secretory and extracellular protein) and cysteine‐rich venom protein (a non‐secretory and extracellular protein; Table 3). We also found other upregulated transcripts, such as prostaglandin reductase and lanC‐like protein, as listed in Table S11.

**TABLE 3:** List of upregulated transcripts in the male macroglands of *Hyloxalus azureiventris* and *Hyloxalus nexipus*, compared to proteins known to be highly expressed in the breeding glands of different frog families.

Gene family	*H. azureiventris*		*H. nexipus*		Frog family
	Arm	Finger IV	Arm	Finger IV	
2D TFPs (e.g., sodefrin precursor‐like factor, phospholipase A2 inhibitor)	—	5.7	9.1	5.4	Hylidae^a^, ^b^
					Pipidae^a^
					Nyctibatrachidae^a^
					Ranidae^c^
					Dendrobatidae^d^
Lysozyme C‐1‐like (LYZ)	—	—	3.9	—	Hylidae^a^
					Ranidae^a^
					Nyctibatrachidae^a^
					Micrixalidae^a^
					Dendrobatidae^d^
Pancreatic lipase (PNLIP)	—	1	—	—	Hylidae^a^
					Dendrobatidae^d^
Trypsin superfamily (e.g., transmembrane protease serine; serine protease like)	5.3	—	—	—	Ranidae^a^
					Dicroglossidae^a^
Cysteine rich secretory proteins (CRISP‐family, e.g., cysteine‐rich venom protein)	—	0.5	1	—	Hylidae^b^
					Ranidae^c^
Ovostatin	—	0.8	0.7	—	Hylidae^b^
Ranaspumin	—	0.9	—	2.8	Dendrobatidae^d^
Cochlin (COCH)	2.3	1.3	5.8	1.8	Dendrobatidae^d^
TIL superfamily (e.g., kunitz‐type serine protease inhibitor; serine protease inhibitor)	5.3	—	1	—	Bufonidae^a^
Serotransferrin (TF)	1.7	—	—	—	Dendrobatidae^d^
Cystatin (CST)	0.8				Ranidae^c^
Tyrosinase (TYR)	—	1.4	—	—	Dendrobatidae^d^

*Note:* Values refer to the log_2_ fold change between the macroglands (target tissue) and control tissue. For proteins encoded by multiple transcripts, only the transcript with the highest log_2_ fold change is shown.

^a^ Bossuyt et al. (2019).

^b^ Schulte et al. (2021).

^c^ Schulte et al. (2022).

^d^ Abondano Almeida et al. (2024).

Notably, we identified a commonly upregulated transcript in the arms and fingers of both species that encodes the cochlin protein, with a relatively high log_2_ fold change (> 1.3) in all target tissues in comparison to control tissues (Table 3), a protein also differentially expressed in the swollen fingers of other dendrobatids (Abondano Almeida et al. 2024). Cochlin is an extracellular protein that is highly abundant in the human cochlea (Lin et al. 2019; Robertson et al. 2006) and is also present in the eye, spleen, thymus, brain and cerebellum (Burgess et al. 2016). It presumably involves hearing and balance functions (Robertson et al. 1998). Additionally, cochlin is reported to be highly expressed in the swimbladder of channel catfish, where it is associated with sound reception (Yang et al. 2018). However, to date, no specific function in reproduction has been identified for this protein in frogs.

Interestingly, we observed a high intraspecific variation in gene expression levels (measured as transcripts per million, TPM) in the integument of the distal end of the upper arm, the BAG area, and the fingers of both *H. azureiventris* and *H. nexipus* (Table S10). This variation was particularly pronounced in *H. nexipus* , where two individuals (1 and 3) exhibited high expression of some SPF transcripts (> 100 TPM), while the other two (2 and 4) showed markedly lower expression levels (< 30 TPM). A similar pattern was observed for other transcripts, such as the Kunitz‐type serine protease inhibitor, where individuals with higher SPF transcript expression also displayed elevated levels (> 400 TPM), whereas the other two had much lower expression (< 120 TPM). A comparable trend was found in the swollen fingers of *H. nexipus* . Notably, individual 1 exhibited the lowest SPF expression across all transcripts detected in the fingers, while individual 3 had the highest expression, followed by individual 2. Most SPF transcripts showed high expression in two individuals (3 and 2, > 220 TPM), whereas the other two (1 and 4) had expression levels below 10 TPM. One particular SPF transcript showed an exceptionally high expression in individual 3 (> 2700 TPM), whereas individual 2 had moderate expression (< 300 TPM) and individuals 1 and 4 showed minimal expression (< 10 TPM). In *H. azureiventris* , the expression pattern was more consistent, with one SPF transcript exhibiting low expression (< 10 TPM) in three out of four individuals, while the remaining individual displayed a higher expression (> 100 TPM).

It is important to note that females were excluded from this study because no specialised glands in either swollen fingers or arms have been reported in females in any of the species examined histologically (Grant et al. 2006, 2017; Cavalcanti et al. 2022). Therefore, it is less likely that differential expression of a putative protein would be detected when the structures responsible for its production and secretion are apparently absent. Nevertheless, our observation of specialised glands and SPF expression in tissues without externally visible glandular structures makes it premature to rule out the presence of hidden glands or similar mechanisms in females, where functional pheromone‐secreting glands might exist.

## 4 Discussion

Our study confirms a notable case of co‐occurrence of swollen fingers and BAGs in *H. nexipus* males. This dual glandular system is unknown in other dendrobatids, making *H. nexipus* one of the few species in the family with two distinct specialised macroglands. While the BAG has been reported in a few species of *Hyloxalus* (Grant and Castro‐Herrera 1998; Grant et al. 2006, 2017), we provide here the first histological analysis of its glandular composition in *H. nexipus* , revealing that it is primarily composed of SSGs.

Cavalcanti et al. (2022) previously examined the finger integument of *H. nexipus* and identified SMGs only in males, at the base of finger IV (coincident with the externally visible swelling) and in fingers II, III and V (without evident swelling). Furthermore, we found that both the SMGs of the fingers and the SSGs of the BAG differentially express SPF, a well‐characterised proteinaceous pheromone system in amphibians (Houck et al. 2007; Janssenswillen, Willaert, et al. 2015; Maex et al. 2016; Van Bocxlaer et al. 2015; Table 4, Figures 5 and 6). While SPF upregulation has been documented in the swollen fingers of two dendrobatid species (Abondano Almeida et al. 2024), its presence in the BAG has not been investigated previously.

**TABLE 4:** Summary of the histological findings and the DE expression of putative pheromones on the male macroglands.

Body location	*Hyloxalus azureiventris*		*Hyloxalus nexipus*	
	Arm	Finger IV	Arm	Finger IV
Gland type	Specialised serous glands	n.d.	Specialised serous glands	Specialised mucous glands
Differential expression of putative pheromone (SPF)	N	Y	Y	Y

Abbreviation: n.d., no data available.

The differential expression of SPF in both glandular structures suggests a role in chemical signalling during courtship and/or mating, expanding our understanding of pheromone production in dendrobatids and indicating that *H. nexipus* may have developed a specialised system for sexual chemical communication within Neotropical poison frogs. The presence of different specialised gland types within the same individual, and even the same male macroglands is not unknown in frogs. For instance, in *Boana* species (Hylidae), the mental gland is composed of both SMGs and SSGs (Brunetti et al. 2015). The co‐occurrence of SMGs in the swollen finger and SSGs in the BAG, combined with the differential expression of SPFs by these two different types of glands, is suggestive of different or potentially complementary roles during reproduction.

Despite the externally visible absence of finger swelling in adult males of *H. azureiventris* , we detected SPF expression in this region. Because finger swelling with SMGs can be subtle or nearly imperceptible in adult males of species like *Allobates talamancae* , *A. tapajos* , *H. anthracinus* and *Leucostethus siapida* (Cavalcanti et al., 2022), it is possible that *H. azureiventris* also has SMGs in its fingers despite the lack of visible swelling. For the moment, this hypothetical suggestion is based on previous research where SPF was upregulated in male finger IV containing SMGs in other dendrobatid species, including *L. brachistriatus* and *E. anthonyi* , linking these glands to pheromone production (Abondano Almeida et al. 2024). However, histological confirmation is required to determine if both sexes of *H. azureiventris* exhibit a similar condition.

### 4.1 SSGs in *Hyloxalus*

Serous glands have been characterised in the dorsal skin of dendrobatid frogs previously (Delfino et al. 2010; De Pérez et al. 1992; Moreno‐Gómez et al. 2014; Neuwirth et al. 1979; Prates et al. 2012; Saporito et al. 2010; Stynoski and O'Connell 2017). In contrast, descriptions of localised serous glands in other body regions in dendrobatid frogs are scarce, with only two species in Hyloxalinae, *Ectopoglossus saxatilis* and *Hyloxalus faciopunctulatus* , reported to possess these glands in the ventral integument (Grant et al. 2017). More recently, serous and mucous glands were identified in the finger and wrist swellings of several species of Aromobatidae and Dendrobatidae, where they function as secondary sexual characteristics of adult males (Cavalcanti et al. 2022). While only OSGs are present, both OMGs and SMGs occur in these macroglands. The SMGs, which contribute to the swelling, are thought to produce sexual pheromones (Abondano Almeida et al. 2024). However, SSGs have not been described previously in dendrobatoids. Our observation of SSGs in the unswollen integument of the distal end of the upper arm in *H. azureiventris* and the BAG in *H. nexipus* add new evidence on the diverse glandular structures within dendrobatid species and their potential role in sexual communication.

The general morphology of the OMGs and OSGs in the unswollen integument at the distal end of the upper arm of *H. azureiventris* and the BAG of *H. nexipus* resembles that described for other dendrobatoids by Cavalcanti et al. (2022). In contrast, the SSGs are hypertrophied, morphologically distinct from OSGs, and highly localised—features consistent with SSGs found in other anuran species (Brunetti et al. 2012, 2015). SSGs are found in other sexually dimorphic macroglands of anurans, such as the mental and lateral glands of hylids, the femoral and shank glands in mantellids, and the iliac glands of cycloramphids. They are classified as sexually dimorphic skin glands (SDSGs; Brunetti et al. 2012, 2015; Gonçalves and de Brito‐Gitirana 2008; Thomas et al. 1993; Vences et al. 2007) and in some species have been shown to produce sexual pheromones (Melnik et al. 2019; Peram et al. 2017).

The SSGs of *H. azureiventris* and *H. nexipus* differ morphologically, with those of *H. azureiventris* possessing a homogeneous syncytium without visible granules under magnification and those of *H. nexipus* possessing a granular syncytium with homogeneous rounded regions. Additionally, the integument of *H. azureiventris* consists of five to seven epithelial cell layers in the epidermis and the stratum compactum is as thick as the stratum spongiosum, whereas that of *H. nexipus* is composed of three cell layers and the stratum compactum is half as thick as the stratum spongiosum in *H. nexipus* , due to the SSGs' size. Similarly, although the BAG of *H. nexipus* was associated with the occurrence of SPF, gene expression analysis did not detect differential expression of SPF or other putative pheromones in the integument of the distal end of the upper arm in *H. azureiventris* (Tables 3 and 4), despite the abundance of SSGs. Further, the lack of histological data from females prevents us from determining whether the SSGs in this species are truly sexually dimorphic.

The SSGs of both species of *Hyloxalus* differ from those in other anurans in a number of characteristics. These include the shape of the secretory portion, the granular or homogeneous aspect of the syncytium, and in some cases, the histochemical properties (Bossuyt et al. 2019; Brizzi et al. 2003; Brunetti et al. 2012, 2015; Delfino et al. 1999; Gonçalves and de Brito‐Gitirana 2008; Luna et al. 2018; O'Donohoe et al. 2019, 2022; Thomas et al. 1993; Vences et al. 2007), likewise with respect to the modified or specialised granular gland of salamanders (Staub and Paladin 1997; Hecker et al. 2003; Wanninger et al. 2018; Rollins and Staub 2017). The only exception is the SSG of the mental gland of the *Aplastodiscus leucopygius* (Hylidae), which resembles in morphology that of the *H. azureiventris* (see Brunetti et al. 2015: figure 3E). Nevertheless, a meticulous comparison is essential to verify the similarities between the glands of these species.

### 4.2 SSG and BAG Evolution in *Hyloxalus*

Although originally proposed as a synapomorphy delimiting the *H. ramosi* group by Grant and Castro‐Herrera (1998), Grant et al. (2017) found that the BAG arose independently at least three times in *Hyloxalus*. *H. azureiventris* and *H. ramosi* are non‐closely related species within the genus, although *H. nexipus* is the sister species of a group of *Hyloxalus* containing *H. azureiventris* (Grant et al., 2027, Anganoy‐Criollo et al., 2022). Our findings that the BAG of *H. nexipus* and the un‐swollen integument at the distal end of the upper arm in *H. azureiventris* contain morphological and transcriptomically distinct SSGs, together with their phylogenetic position, suggest that these SSGs are not homologous but instead represent independent evolutionary origins, as has been proposed for the BAG (Grant et al. 2017). Nevertheless, if the presence of such glands in other species closely related to *H. azureiventris* is demonstrated, it would indicate that they are more widespread and potentially synapomorphic for the clade including *H. nexipus* and *H. azureiventris* . In this scenario, rather than arising entirely independently multiple times, the BAG would simply represent a hypertrophied condition of a more general state, as has been suggested to explain at least some of the apparent independent origins of finger IV swelling (Cavalcanti et al. 2022; Grant et al. 2017). Histological and transcriptomic evidence, and phylogenetic analysis are required from additional species to fully elucidate the evolution of the BAG and related SSGs in *Hyloxalus*.

### 4.3 Macrogland Composition and Gene Expression

The presence of SPF genes in association with both the SMGs of swollen fingers and SSGs of the BAG suggests either a single gene broadly expressed across these glandular structures or distinct SPF genes specialised for each tissue. In plethodontid salamanders, SPF is encoded by multiple genes, with some exhibiting gland‐specific expression while others are distributed across different pheromone‐secreting tissues (Herrboldt et al. 2021). For instance, certain SPF genes are restricted to the mental gland, whereas others are predominantly expressed in the caudal gland, suggesting that different pheromone genes may have been co‐opted into specific gland types over evolutionary time (Herrboldt et al. 2021). However, some SPF genes show broader expression patterns across multiple glandular structures within and between species, as reported in *Notophthalmus viridescens* , where the same SPF genes are expressed in both cheek and cloacal glands (Janssenswillen, Willaert, et al. 2015). Similarly, *H. nexipus* exhibits SPF expression in both the fingers and BAG, highlighting a parallel case of multiple glandular structures contributing to chemical communication. Nevertheless, whether these glands express the same SPF gene or distinct copies specialised for each tissue in *H. nexipus* remains unknown. Notably, the SPFs expressed in the swollen fingers of *L. brachistriatus* and *E. anthonyi* belong to the SPF beta clade (Abondano Almeida et al. 2024), which functions as a pheromone in salamanders (Van Bocxlaer et al. 2015) and is suspected to play a similar role in frogs (Bossuyt et al. 2019). A phylogenetic analysis of SPFs from swollen fingers and BAGs across dendrobatids would help clarify whether this beta clade association is a broader pattern, shedding light on the diversification and potential gland‐specific recruitment of SPF genes.

Furthermore, whether SSGs evolved specifically for protein pheromone secretion remains uncertain. The nature of these glands in the arm varies even among *Hyloxalus* species, further complicating our understanding of their functional role and evolutionary history. Similar functional diversity is seen in the toxic newt *Taricha granulosa* , where tetrodotoxin (TTX), typically linked to poison glands (serous or granular glands), is also found in mucous glands—at higher concentrations in the mucous glands of low‐toxicity populations (Mailho‐Fontana et al. 2019). This challenges the notion that mucous glands solely produce mucus and points to broader glandular roles in amphibian skin (Mailho‐Fontana et al. 2019). In line with this, our results, along with the detection of SPF expression in the male ventrolateral macrogland of the hylid frog *P. macrotympanum* (Schulte et al., 2024), support a more widespread role for serous glands in pheromone production. Moreover, the observed differences in gland composition and gene expression between the BAG and the arm integument of *H. nexipus* and *H. azureiventris* , respectively, indicate functional divergence rather than a conserved role across taxa. Only a few differentially expressed transcripts are shared between species such as cochlin, serine protease inhibitor and myosin‐binding protein (Table 3, Table S10). A similar pattern is evident in the finger integument, where only a few transcripts overlap, including SPF, cochlin, ranaspumin and prostaglandin reductase 1 (Table 3, Table S10), reinforcing the idea that gland function is shaped by species‐specific evolutionary pressures rather than strict gland type.

Additionally, we observed a high intraspecific variation in the gene expression of some transcripts of the BAG and the integument of the distal end of the upper arm, and fingers in both *H. azureiventris* and *H. nexipus* , which could have impacted the gene expression pattern results and levels of expression including the putative protein pheromone. The observed variation in transcript levels across individuals could be related to reproductive condition and potentially reflect differences in gland swelling or developmental stage, which would contribute to variability in expression patterns (TPM values). Since RNA‐seq captures gene expression at a specific point in time, individual differences in reproductive status likely impact the expression of differentially expressed (DE) transcripts including putative pheromones. For this reason, we used four individuals of each species. Some individuals might have been in an active reproductive state, exhibiting fully developed glands and associated proteins or secretions, while others were likely in a non‐reproductive phase, leading to reduced overall expression. Given that male macrogland development is hormonally regulated (for review, see Luna et al. 2018) fluctuations in androgen levels—potentially linked to age or seasonal cycles—may affect gland maturation and pheromone production. Furthermore, reproductive synchrony may be more difficult to maintain in captivity, because environmental cues that regulate natural breeding cycles, such as seasonal changes, are absent; thus, laboratory conditions can disrupt this synchronisation, potentially affecting gland development and pheromone production. More importantly, even when macroglands remain present, pheromone levels can decline significantly during gland regression, a phenomenon that has been documented in other species (Wilburn and Feldhoff 2019; Willaert et al. 2013) and may explain why glands are present but lack pheromone expression.

### 4.4 Behaviour

Histological, histochemical and molecular evidence, along with courtship observations, support the role of swollen fingers in cephalic amplexus—a proposed pheromone transmission mechanism in dendrobatoids (Abondano Almeida et al. 2024; Cavalcanti et al. 2022; Grant et al. 2017). However, the function of the BAG remains less clear. The limited natural history data for most *Hyloxalus* species, including *H. nexipus* (Grant et al., 2006; Quiguango‐Ubillús and Coloma, 2008), make it difficult to determine whether this macrogland is associated with courtship behaviour. Nonetheless, Grant and Castro‐Herrera (1998; see also Grant et al. 2006) hypothesised that the BAG plays a role in reproductive amplexus, as it is absent in juveniles and females and appears to be exaggerated in sexually active males, analogous to the swollen finger. Our results on pheromone gene expression and presence of SSGs in the BAG of *H. nexipus* also point to a role of this macrogland in reproduction, corroborating the hypothesis of Grant and Castro‐Herrera (1998). In addition, if amplexus is confirmed in this species, the BAG may contribute to the nuptial embrace, whereby glandular tissues in the fingers and arms make contact with the female's body and head, potentially transferring SPF pheromones simultaneously or sequentially, either transdermally or directly to sensory organs such as the olfactory system. Given the lack of behavioural data, we cannot rule out its involvement in other reproductive stages including pre‐ and post‐nuptial interactions or other tactile behaviours.

Conversely, *H. azureiventris* exhibited differential SPF expression in the fingers but not in the unswollen integument of the distal end of the upper arm. Although this species lacks amplexus (Lötters et al. 2000), tactile interactions still occur during mating, with males touching the female's dorsum during oviposition and sometimes positioning their arms around the female's body without fully embracing her (Lötters et al. 2000, 2007; K.‐H. Jungfer, pers. comm.). This hand‐to‐back contact could enable SPF transmission via the fingers, potentially transdermally, as the fingers do not reach sensory organs such as the olfactory system. However, this hypothesis requires experimental validation. Moreover, as mentioned, the lack of differential SPF expression in the arm makes a reproductive function questionable. Thus, the function of the SSGs in the integument of the distal portion of the upper arm in *H. azureiventris* remains unclear, underscoring the need for further investigation.

In dendrobatoid frogs, the loose nature of cephalic amplexus allows females to disengage freely at any stage of courtship, even after reaching the oviposition site. This highlights the potential importance of chemical signals, particularly SPFs, which are hypothesised to induce or accelerate oviposition, reducing courtship duration and associated predation risks (Schulte et al. 2022). SPF could also maintain females physiologically primed for oviposition after disengagement, ensuring timely egg‐laying and maximising fertilisation success. In territorial species (common in dendrobatoids; for review, see Pröhl 2005), males guide females to suitable oviposition sites within their territories, coupling chemical communication, physiological priming and territoriality, to maximise fertilisation success, highlighting the interplay between sexual selection and the evolution of specialised glands and their pheromone secretions.

Our study sheds light on the complexity of chemical communication in Dendrobatidae by revealing tissue‐ and species‐specific expression of putative pheromones in specialised glands including the swollen fingers and the black arm gland. The identification of specialised serous glands and the likely breeding function of the black arm gland in *H. nexipus* supports the role of glandular pheromone expression in amphibian reproduction. These findings highlight the functional diversity of sexually dimorphic glands and underscore the need for further research on the molecular evolution of pheromone‐related genes in Neotropical poison frogs.

## Author Contributions

D.A.A. and L.M.S. designed the study. D.A.A., M.A.‐C., S.K. and T.G. acquired the data. D.A.A., M.A.‐C., S.K. and T.G. analysed and interpreted the data. D.A.A., M.A.‐C., T.G. and L.M.S. wrote the manuscript.

## Conflicts of Interest

The authors declare no conflicts of interest.

## Supporting information


**Table S1:** mec70162‐sup‐0001‐TablesS1‐S11.zip.
**Table S2:** mec70162‐sup‐0001‐TablesS1‐S11.zip.
**Table S3:** mec70162‐sup‐0001‐TablesS1‐S11.zip.
**Table S4:** mec70162‐sup‐0001‐TablesS1‐S11.zip.
**Table S5:** mec70162‐sup‐0001‐TablesS1‐S11.zip.
**Table S6:** mec70162‐sup‐0001‐TablesS1‐S11.zip.
**Table S7:** mec70162‐sup‐0001‐TablesS1‐S11.zip.
**Table S8:** mec70162‐sup‐0001‐TablesS1‐S11.zip.
**Table S9:** mec70162‐sup‐0001‐TablesS1‐S11.zip.
**Table S10:** mec70162‐sup‐0001‐TablesS1‐S11.zip.
**Table S11:** mec70162‐sup‐0001‐TablesS1‐S11.zip.

## Data Availability

The raw reads generated and analyzed during the current study have been deposited in the European Nucleotide Archive (ENA) under project accession number PRJEB102072. A fasta‐file with the RNA‐seq assemblies are available on Dryad (https://doi.org/10.5061/dryad.vq83bk45g).
