# Supplementary material for: Histological and Gene Expression Analyses of the Arm and Finger Macroglands of Two Hyloxalus Frogs (Dendrobatidae)
Source: Mol Ecol. 2025 Nov 6;34(22):e70162. doi: 10.1111/mec.70162 (PMC12617036; doi:10.1111/mec.70162)
Supplement: Supplementary file 1 — Table S1: mec70162‐sup‐0001‐TablesS1‐S11.zip. Table S2: mec70162‐sup‐0001‐TablesS1‐S11.zip. Table S3: mec70162‐sup‐0001‐TablesS1‐S11.zip. Table S4: mec70162‐sup‐0001‐TablesS1‐S11.zip. Table S5: mec70162‐sup‐0001‐TablesS1‐S11.zip. Table S6: mec70162‐sup‐0001‐TablesS1‐S11.zip. Table S7: mec70162‐sup‐0001‐TablesS1‐S11.zip. Table S8: mec70162‐sup‐0001‐TablesS1‐S11.zip. Table S9: mec70162‐sup‐0001‐TablesS1‐S11.zip. Table S10: mec70162‐sup‐0001‐TablesS1‐S11.zip. Table S11: mec70162‐sup‐0001‐TablesS1‐S11.zip. [file MEC-34-e70162-s001.zip › mec70162-sup-0001-TablesS1-S10.pdf]

MOLECULAR ECOLOGY

Supplemental Information for:

Histological and Gene expression analyses of the arm and finger macroglands of two *Hyloxalus* frogs (Dendrobatidae)

Diana Abondano Almeida, Marvin Anganoy-Criollo, Taran Grant, Sofiia Klimovych, Lisa M. Schulte

MOLECULAR ECOLOGY

TABLE S1. Specimen data for the wild-caught *Hyloxalus nexipus* male used in the histological analysis, deposited in the Herpetology Division of the University of Kansas Natural History Museum.

Hyloxalus nexipus	Peru	14 km ESE Shapaja, San Martin	KU 211827

TABLE S2. Read statistics of the RNAseq samples from *Hyloxalus azureiventris* paired sample **arm** and **leg** tissue

	**Male 1 - arm**	**Male 1 - leg**	**Male 2 - arm**	**Male 2 - leg**	**Male 3 - arm**	**Male 3 - leg**	**Male 4 - arm**	**Male 4 - leg**
Raw sequencing reads (pairs)	25,309.055	32,170.589	31,267.072	32,363.192	30,421.654	29,842.807	24,109.971	20,362.860
Reads passing trimming & error correction	24,358.574	30,816.766	30,080.632	31,209.566	29,255.882	28,642.251	23,242.620	19,873.373
Hisat2 overall alignment rate	77.99%	78.20%	79.29%	78.28%	80.32%	78.52%	79.63%	80%

TABLE S3. Read statistics of the RNAseq samples from *Hyloxalus azureiventris* paired sample **fingers** and **toes**

	**Male 1 – finger IV**	**Male 1 - toe**	**Male 2 - finger IV**	**Male 2 - toe**	**Male 3 - finger IV**	**Male 3 - toe**	**Male 4 - finger IV**	**Male 4 - toe**
Raw sequencing reads (pairs)	22,589.714	19,099.216	19,668.242	20,048.204	21,738.853	22,018.327	32,788.408	20,924.078
Reads passing trimming & error correction	21,372.436	18,421.118	18,918.947	19,491.131	20,862.224	21,301.432	31,802.352	20,457.576
Hisat2 overall alignment rate	78%	79.32%	66.66%	75.55%	78.08%	78.99%	80.66%	80.89%

MOLECULAR ECOLOGY

TABLE S4. Read statistics of the RNAseq samples from *Hyloxalus nexipus* paired sample **arm** and **leg** tissue

	**Male 1 - arm**	**Male 1 - leg**	**Male 2 - arm**	**Male 2 - leg**	**Male 3 - arm**	**Male 3 - leg**	**Male 4 - arm**	**Male 4 - leg**
Raw sequencing reads (pairs)	24,172.924	28,703.465	41,651.897	23,698.949	20,276.642	24,539.002	29,363.503	18,092.776
Reads passing trimming & error correction	23,280.851	27,523.463	40,189.890	22,690.351	19,447.454	23,613.705	28,456.250	17,732.014
Hisat2 overall alignment rate	83.49%	83.67%	81.89%	82.87%	82.03%	81.61%	83.20%	81.67

TABLE S5. Read statistics of the RNAseq samples from *Hyloxalus nexipus* paired sample **fingers** and **toes**

	**Male 1 - swelling**	**Male 1 - toe**	**Male 2 - swelling**	**Male 2 - toe**	**Male 3 - swelling**	**Male 3 - toe**	**Male 4 - swelling**	**Male 4 - toe**
Raw sequencing reads (pairs)	17,469.084	18,225.725	23,720.366	17,425.896	34,977.352	20,458.872	19,082.423	36,930.758
Reads passing trimming & error correction	16,618.881	17,752.864	23,093.113	16,763.305	33,867.871	19,704.649	18,290.042	35,860.998
Hisat2 overall alignment rate	83.99%	83.36%	81.65%	80.33%	83.02%	81.71	82.98%	67.12%

MOLECULAR ECOLOGY

TABLE S6. De novo transcriptome assembly paired sample **arm** and **leg** tissue statistics of *Hyloxalus azureiventris*

n transcripts in assembly	279295
Longest transcript (bp)	83172
Mean transcript length	655.85
n transcripts over 1,000 bp	42140
n transcripts over 10,000 bp	93
n transcripts with ORF	29882
Mean ORF %	48.16
N50	941
BUSCO % complete	88.6
BUSCO % duplicated	2.3
BUSCO % fragmented	3
BUSCO % missing	8.4
n transcripts annotated	51473

TABLE S7. De novo transcriptome assembly paired sample **fingers** and **toes** statistics of *Hyloxalus azureiventris*

n transcripts in assembly	312416
Longest transcript (bp)	17738
Mean transcript length	579.9
n transcripts over 1,000 bp	36342
n transcripts over 10,000 bp	100
n transcripts with ORF	27680
Mean ORF %	50.12
N50	726
BUSCO % complete	88.6
BUSCO % duplicated	2.1
BUSCO % fragmented	3.1

MOLECULAR ECOLOGY

BUSCO % missing	8.3
n transcripts annotated	48740

TABLE S8. De novo transcriptome assembly paired sample **arm** and **leg** tissue statistics of *Hyloxalus nexipus*

n transcripts in assembly	230364
Longest transcript (bp)	74188
Mean transcript length	673.25
n transcripts over 1,000 bp	35821
n transcripts over 10,000 bp	83
n transcripts with ORF	25795
Mean ORF %	48.62
N50	1012
BUSCO % complete	89.7
BUSCO % duplicated	2
BUSCO % fragmented	2.4
BUSCO % missing	7.9
n transcripts annotated	43582

TABLE S9. De novo transcriptome assembly paired sample **fingers** and **toes** statistics of *Hyloxalus nexipus*

n transcripts in assembly	273611
Longest transcript (bp)	19303
Mean transcript length	567.29
n transcripts over 1,000 bp	27161
n transcripts over 10,000 bp	37
n transcripts with ORF	52377
Mean ORF %	66.46

MOLECULAR ECOLOGY

N50	595
BUSCO % complete	77.1
BUSCO % duplicated	1.8
BUSCO % fragmented	9.5
BUSCO % missing	13.4
n transcripts annotated	63325

TABLE S10. List of annotated genes and differential expression analysis of *H. azureiventris* and *H.* nexipus (Excel tables), which will be archived subsequently to Dryad

MOLECULAR ECOLOGY

TABLE S10. List of selected differentially expressed upregulated genes in the male macroglands of *H. azureiventris* and *H. nexipus*. Values below each tissue type refer to the log fold$_2$ change between the target and control tissue (in the case of several transcripts coding for a protein, the highest log fold was chosen)

Gene	Secretory / Extracellular	*Hyloxalus azureiventris*		*Hyloxalus nexipus*	
		**Arm swelling**	**Finger IV**	**Arm swelling**	**Finger IV**
		**Specialized serous glands**	*	**Specialized serous glands**	**Specialized mucous glands**
Glycine-rich cell wall (GRP)	Y	1.17	–	–	–
Uromodulin (UMOD) Tamm–Horsfall protein (THP)	Y	–	–	1.9	–
di-N-acetylchitobiase (CTBS)	Y	–	–	1.4	–
matrix Gla protein (MGP)	Y	–	–	1	–
myosin-binding protein (MYBP)	N	4.9	–	2	–
pG1 protein	N	3.7	–	–	–
ADP-ribosylation factor (ARF)	N	–	–	3.1	1.8
actin, alpha cardiac muscle 1 (ACTC1)	N	–	–	1	–
glutamine synthetase (GSS)	N	–	–	0.8	–
alcohol dehydrogenase 1 (ADH)	N	–	0.2	0.7	–
pancreatic triacylglycerol lipase-like (PNLIP)	N	–	1	–	–
lanC-like protein (LANCL2)	N	–	0.5	–	–
NADH dehydrogenase	N	–	0.3	–	–
prostaglandin reductase 1 (PTGR1)	N	–	2.8	–	2

*Unknown whether specialized glands are present
